# System for Face Recognition under Different Facial Expressions Using a New Associative Hybrid Model Amαβ-KNN for People with Visual Impairment or Prosopagnosia

**DOI:** 10.3390/s19030578

**Published:** 2019-01-30

**Authors:** Moisés Márquez-Olivera, Antonio-Gustavo Juárez-Gracia, Viridiana Hernández-Herrera, Amadeo-José Argüelles-Cruz, Itzamá López-Yáñez

**Affiliations:** 1 CICATA Unidad Legaria, Instituto Politécnico Nacional, Av. Legaria No. 694 Col. Irrigación, CDMX 11500 Mexico City, México; mmarquezo@ipn.com; 2 CIITEC, Instituto Politécnico Nacional, Cerrada Cecati s/n Col. Sta. Catarina, Azc., CDMX 02250 Mexico City, México; 3 CIC, Instituto Politécnico Nacional, Av. Juan de Dios Bátiz, Esq. Miguel Othón de Mendizábal, Col. Nueva Industrial Vallejo, CDMX 07738 Mexico City, México; 4 CIDETEC, Instituto Politécnico Nacional, Av. Juan de Dios Bátiz, Esq. Miguel Othón de Mendizábal, Col. Nueva Industrial Vallejo, CDMX 07700 Mexico City, México; ilopezy@ipn.mx

**Keywords:** face recognition, assistive technologies, facial expressions, impaired vision, alpha-beta associative memories, correlation matrix, k-nearest neighbors (KNN), associative memory

## Abstract

Face recognition is a natural skill that a child performs from the first days of life; unfortunately, there are people with visual or neurological problems that prevent the individual from performing the process visually. This work describes a system that integrates Artificial Intelligence which learns the face of the people with whom the user interacts daily. During the study we propose a new hybrid model of Alpha-Beta Associative memories (Amαβ) with Correlation Matrix (CM) and K-Nearest Neighbors (KNN), where the Amαβ-CMKNN was trained with characteristic biometric vectors generated from images of faces from people who present different facial expressions such as happiness, surprise, anger and sadness. To test the performance of the hybrid model, two experiments that differ in the selection of parameters that characterize the face are conducted. The performance of the proposed model was tested in the databases CK+, CAS-PEAL-R1 and Face-MECS (own), which test the Amαβ-CMKNN with faces of subjects of both sexes, different races, facial expressions, poses and environmental conditions. The hybrid model was able to remember 100% of all the faces learned during their training, while in the test in which faces are presented that have variations with respect to those learned the results range from 95.05% in controlled environments and 86.48% in real environments using the proposed integrated system.

## 1. Introduction

It is estimated that more than a billion people in the world live with some type of disability; that is, around 15% of the population, additionally, according to the World Health Organization (WHO) statistics [1] the percentage of people with disabilities is growing. This is mainly due to the aging of a sector of the population and the increase in chronic degenerative problems, hence the importance and impact of developing technological tools that improve aspects such as [2]: Rehabilitation, assistance, development of environments, education, and insertion into employment, among others. The continuous technological advances represent an opportunity to adapt environments that allow the development of people with disabilities. For this, it is necessary to know the needs that each of the disabilities represents, whether they are cognitive, motor, auditory, visual or mixed.

Inclusion is one of the great challenges [3,4,5] for this, it is necessary the adaptation of the environment [6] or the creation of implements that allow the individual with some disability an easy interaction with the different elements around him [7,8,9], being the option to incorporate implements that are convenient due to the associated costs. Leo et al. [10] use the term “Assistive Technologies” (AT) to refer to all those technological developments focused on improving the condition of people who suffer from some type of disability, this from a point of view based on “user-need oriented”, they present an interesting analysis of the advances in sensors, electronics and other devices that make use of artificial vision [11,12,13] to overcome the functional limitations and improve the quality of life of the individuals, concluding that the AT aimed at the location, detection of objects and individuals are those that present greater versatility with respect to problems related to mental functions, personal mobility, sensory functions and daily activities.

According to the WHO in 2010, there are 285 million people in the world with total or partial visual impairment, which represents 4.25% of the total population [14]; this is the reason why the scientific community shows an interest in proposing alternatives that facilitate the interaction and interpretation of the navigational environment of the individual, where it is necessary to consider that the technology should be easily adopted by the users [15]. Fernandes et al. [16] present an interesting review of recent technologies for the special assistance and orientation of people who are visually impaired. Rabhi et al. [17] propose performing recognition of facial expressions and using them as command modes for wheelchair control, where they use neural networks programmed into Raspberry Pi and an application installed on a Smartphone to capture the image and human-machine interaction, the results obtained range from 95% to 97.1% effective. Nandini and Seeja [18] present a new algorithm for easy navigation in a supermarket for visually impaired people, the results show that the algorithm was able to find the optimal path avoiding obstacles and minimizing distance and turns in the route. Dunai et al. [8] approach the problem faced by blind people when trying to recognize Euro banknotes of different denominations, for that they propose to implement recognition algorithms in a Raspberry Pi and a compatible camera, with which they achieve between 97.5% recognition. Huang et al. [19] propose wearable tactile traffic lights assistive device (WTTLAD) to help safely cross blind or visually impaired people, whose accuracy rate in the field reached 96.67%. Google also ventures into the development of technologies with augmented reality with the Tango project [20,21,22], which although it is not exclusive for use in people with some type of disability, since the project aims to create a portable artificial vision tool to map 3D spaces with a mobile device, therefore it has interesting applications that are being adapted for people with visual problems. As in the work presented by Jafri et al. [23] who present a system that integrates infrared technology to assist visually impaired users in detecting obstacles in their path while independently navigating indoors, the system has been introduced in Google Project Tango Tablet Development Kit.

### Background and Scope of This Study

The recognition of faces is a process that is naturally performed by human beings from their earliest years, it is a tool for survival and evolution because it allows the recognition of other people and the use of memories about those; unfortunately, for people who suffer from visual disability or prosopagnosia [24] the process of face recognition is complicated, since they are unable to identify by visual means a known or unknown person, hence the importance of proposing a device that performs the process automatically and tells the user the identification of the person in front of them. For this it is important to consider that the natural process performed by humans is complex and robust, since it is necessary to extract, analyse, discriminate, and classify a set of data provided by the sense of sight. Consequently, automating facial recognition requires intensive computational processing. Technological advances and a wide range of applications have motivated the scientific community to work on developing systems with the ability to performing this process automatically. The process of automatic face recognition can be divided into two subproblems: “Face detection” and “facial recognition”.

The aim of face detection is to find a face within an image. Cho et al. [25] direct their efforts to in the detection of faces at night using a single visible-light camera and a convolutional neural network (R-CNN) obtaining a precision of 90% in low lighting conditions. Ma et al. [26] use a thermal camera and the method of local features for face detection, they also propose an extension of Multi-Block LBP, with these features, an AdaBoost-based cascade is trained, comparing the performance of the proposed algorithm with other cascade classifiers training them with different characteristics, where the competitiveness of the model is demonstrated. Wu et al. [27] propose the use of convolutional neural networks (CNNs) for the detection of faces and for the estimation of the pose, explaining that if a CNN is used for each process the performance is committed since they are independent networks. Therefore to solve the problem they propose a multi-task CNN cascade framework that integrates these two tasks in a single network, additionally, the fusion of features is used to improve performance.

The second subproblem facial recognition focuses is determining who owns the faces previously found. He et al. [28] approach the problem of face recognition with occlusions, proposing an approach called dynamic feature matching (DFM) which combines fully convolutional networks and sparse representation classification (SRC) to address partial face recognition, regardless of the various face sizes. Results show that DFM performance is higher than partial face recognition techniques reported in the literature. If the face recognition system must be designed to work in real conditions, then it is necessary to address adjacent problems such as changes in illumination. Yu-Feng et al. [29] use a discriminative multi-layer system to separate the reflective component and lighting effects integrated into the image, while Hu et al. [30] work with illumination normalization based on the high-and low-frequency components decomposed.

Other works address the problems of illumination and pose at the same time. Thus, if we consider the real conditions encountered by a face recognition system, it is necessary to consider that people perform facial gestures as a way to enrich communication. This results in a deformation in the face, which generates a new problem because the recognizer must be able to determine who a face belongs to, regardless of whether the target person expresses joy, anger, sadness, or another emotion.

This study aims to develop a facial recognition system in real time for people with visual impairment or prosopagnosia who have limitations when trying to identify people visually, thus it is proposed that the system is able to recognize the face of a person. The system integrates a plastic structure mounted on the head that integrate a camera, which sends video to be processed in a mini PC Jetson TK1 developed by NVIDIA (Beijing, China), where a Haar Feature-based cascade classifiers with AdaBoost for face detection algorithm was implemented, then the active appearance model is used to extract 42 characteristic points of the face, and finally, we propose to use a new hybrid model Amαβ-CMKNN as a classifier to determine to whom a particular face belongs. The hybrid model is trained with characteristic biometric vectors extracted from images from the Cohn-Kanade plus (CK+) database [31], which includes sequences of images with different facial expressions.

This paper is organized as follows: Section 2 provides a general description of proposed system and the theoretical basis of the models and techniques used in this research. Section 3 presents a detailed description of the two experiments used to demonstrate the competitiveness of the proposed model, as well as the results obtained in both experiments. Section 4 describes the integration and adaptation of the system to work under real conditions. Finally, in Section 5, the conclusions and discussion are given.

## 2. Materials and Methods

**Figure 1:**
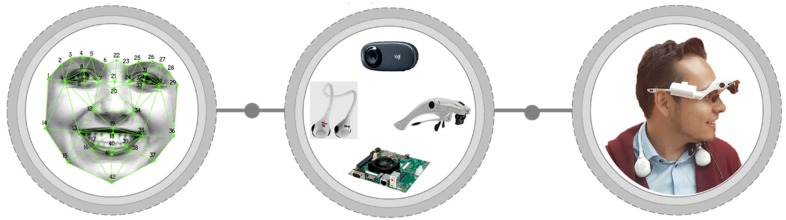
Processes for the development of the face recognition system for people with visual disabilities, (**a**) face recognition under different facial expressions, (**b**) implement the devices and adapt them to people with disabilities, (**c**) system integration.

The development of the proposed system is divided into three processes shown in Figure 1, the first process is to propose, design and implement algorithms to recognize faces even when they present different facial expressions, the second process focuses on the search and selection of compatible devices that are at the same time comfortable for use by a person with visual impairment or prosopagnosia. Finally, we propose the integration phase where the aim is that the algorithms for facial recognition that were previously programmed, in a conventional personal computer (PC), are modified to work with the adapted devices thus that the individuals are able to use them in an easy way. The following subsections describe each process in detail.

### 2.1. Face Recognition under Different Facial Expressions

**Figure 2:**
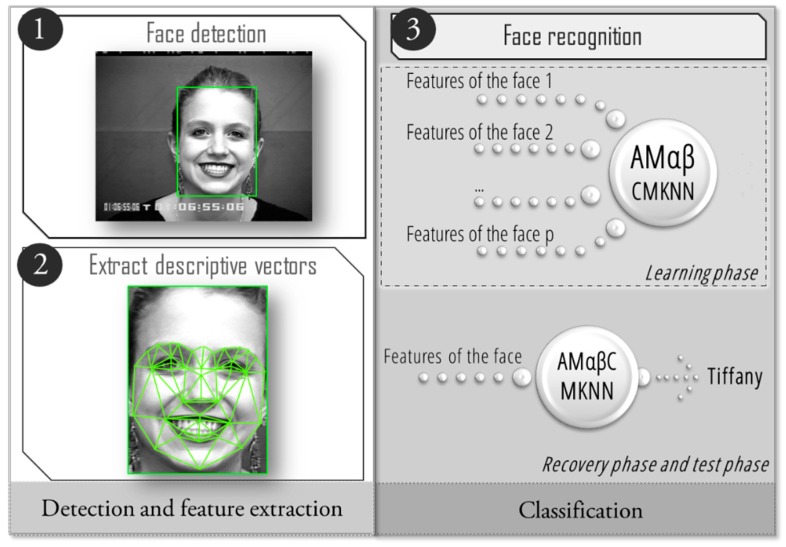
General process for face recognition using Amαβ- correlation matrix k-nearest neighbor (CMKNN).

The facial recognition process has been divided into three steps, as illustrated in Figure 2, the first step focuses on finding a face within an image to segment it from the rest of the image; once the face has been located the next step is to extract descriptive vectors that characterize the detected face. It is necessary to obtain the vectors of each image of the training set, as they will be learned by the associative memories Alpha-Beta CM-KNN, which is the classifier proposed in this study to determine to whom a given face belongs.

### 2.2. Detecting Faces Using Haar-Like Features (HLF)

HLF Cascade is used to detect a face within an image, which is a fast and robust classifier proposed by Viola and Jones [32], HLF has been widely used in the current literature for the detection of faces [26,33,34,35]. The algorithm is based on the extraction of a set of features called Haar-like features, in which rectangular areas that are divided into one or more parts are considered. Each feature is superimposed on the image in all positions and all possible sizes, with the purpose of summing the intensities of the pixels in the area in black and subtracting them from the sum of the intensities of the pixels in the white sector. To quickly calculate each of the rectangles, we use a representation of an image called an integral image (ii), which consists of the sum of the pixels above and to the left of a reference point (x,y).

(1) $$ii_{\left(x,y\right)}=\underset{x^{\prime }\leq x,y^{\prime }\leq y}{\sum }i\left(x^{\prime },y^{\prime }\right)$$

To use the HLF as a weak classifier, one must consider x a region of an image associated with the value of the HLF, where HLF(x) results from calculating the HLF in the x region, and θ is the threshold. Unfortunately, this classifier is not precise. Therefore, we proposed to use the AdaBoost (adaptive boosting) technique to construct a strong classifier. For this, it is necessary to make a linear combination of the HLF weak classifiers. H(x) is a strong classifier and $h_{t}\left(x\right)\to \left\{-1,1\right\}$ represents each of the HLF weak classifiers.

(2) $$H\left(x\right)=sign\left(\overset{T}{\underset{t=1}{\sum }}\alpha_{t}h_{t}\left(x\right)\right)$$

Finally, to make the algorithm more efficient, we use the classifier in a cascade structure, thus creating a binary decision tree, where each node is replaced by a trained AdaBoost classifier, with the objective of limiting the number of features computed by each window.

### 2.3. Feature Extraction with Active Appearance Model (AAM)

AAM has been widely used in the current literature [36,37,38,39,40], in this paper, AAM [41] is used to locate the points of interest within the faces that have previously been segmented, in addition, a geometric model is used to align the face and determine the pose of the face in case the face is not in front of the camera. The shape s in AAM is represented as points joined forming a mesh where $s=(x_{1},y_{1},x_{2},y_{2},\ldots ,x_{n},y_{n})$ with dimension ${ℜ}^{k}$, thus that if the images in this study are 2D, then k=2. Therefore, since for this study 42 representative points are proposed, and since AAM allow linear shape variation, the shape s can be expressed as a base shape $s_{0}$ plus a linear combination of m shape vectors $s_{i}$.

(3) $$s=\tau \left(s,\;R,\;T;s_{0}+\overset{m}{\underset{i=1}{\sum }}p_{i}s_{i}\right)$$

In this expression, the coefficients ${\left(p_{1},p_{2,}\ldots ,p_{m}\right)}^{T}$ are the parameters of the forms [36]. Obtaining a vector s that represents the form and whose length is 2n, it follows that the length of the vector for this study is s  = 2 × 42 = 84. While, τ(s,R,T) is a rigid transformation that performs a rotation by the rotation matrix $T={\left[t_{x},\;t_{y}\right]}^{T}$, i.e., if τ(s,R, T) is applied to a point ${\left[x,\;y\right]}^{T}$:

(4) $$\tau \left(s,\;R,\;T;\;{\left[x,\;y\right]}^{T}\right)=sR\left[\begin{array}{c} x\\ y \end{array}\right]+T$$

Subsequently, to reduce the dimension of the data, the technique the Analysis of Principal Components (PCA) is used, therefore, the $\sum_{i=1}^{m}s_{i}$ is done with a PCA and the training set forms. The model is defined as an orthogonal linear transformation that projects data into a new coordinate system defined by the coordinate axes of the variances. The dimension of the data are reduced by selecting only the data that best align with the variance and discarding the rest of the data. To train the mesh with PCA, the vectors $s_{0}$ are considered to represent the eigenvectors corresponding to the n vectors’ eigenvalues. The training shapes are normalized by an iterative Procrustes analysis to remove global variations.

In this work we chose to align each of the s samples with each other in relation to the position of the eyes and the columella´s base of the nose [42], the vertex of the mean shape representing the position of the columella is positioned on the horizontal center of the image and at 1/3 of the height of the image, thus due to the morphology of the face it is known that the eyes are localized on the lower margin of the nasal septum. Once the eyes are located, the images are rotated to set the eyes on the same horizontal line, for this an initial pattern is selected and the scale, rotation, and translation with respect to the frontal position is calculated. For this, an imaginary line between the center points of each eye, left and right, after the angle of inclination and scale is calculated [36].

### 2.4. Associative Memories (AM)

Associative Memories (AM) have their inspiration in human memory. Consequently, the technique’s competitive advantage resides in storing information that can be recovered efficiently. New lines of research have emerged with the aim of improving the models [43,44,45,46], which resulted in better performance when recalling previously learned patterns, but at the same time allowed them to associate altered patterns by an additive, subtractive or combined noise that was not presented to memory during their learning. Ritter and Sussaner [47] present the morphological memories which return the qualities of the models of the classic memories, integrating the concepts of dilatation and erosion of mathematical morphology. Consequently, the morphological memories make use of the maximum (⋁) and minimum (⋀) operators, which results in the auto-associative type being able to store and perfectly recover any number of learned patterns.

### 2.5. Hybrid Model Amαβ-CMKNN

The proposed model aims to improve the performance of Associative memories Alpha-Beta in the task of classification of patterns with noise with which it has not been previously trained, thus we propose to integrate a correlation matrix (CM) and the k-nearest neighbors (KNN) method, to ensure that the memory relates more precisely to the output patterns generated when trying to associate the incoming pattern with a learned pattern. In the first part of this section, the proposed model is described in detail, where it begins with the associative memories Alpha-Beta (Amαβ) and later the integration of the CMKNN in the memory.

The associative memories Alpha-Beta (Amαβ) [48], which returned to the fundamental principles of the operators of max (⋁) and min (⋀) of the morphological memories. It allows them to recover all the patterns of the training set However, its contribution is based on the inclusion of two new operators, alpha (α) and beta (β), used in the recovery and classification of patterns. The incorporation into the model of α and β makes it possible for the memory to also be able to classify patterns that have not previously been learned. At this point, it became a model of supervised learning used in problems of recognition and classification.

The Amαβ is characterized by including the binary operators α and β, where the α operator is used for the learning phase and the β operator is used during the recovery phase. For the definition of the binary operators, we have the sets A and B, which contain the elements:

A={0,1}   and B={0,1,2}

Based on sets A and B the binary operator α is defined as A α A→B. The operator β is given by B β A→A. The mathematical demonstration and algebraic properties of both operators is described in the work of Marquez [48].

### 2.6. Phases of Amαβ

The Amαβ is a supervised learning model. Therefore, they make use of a database with patterns that represent the most significant features that define the objects that are to be recognized. Additionally, the pattern are properly cataloged in classes. Validation methods such as the k-fold cross validation, bootstrap, or leave-one-out are used to segment the database into two parts. The first part of the patterns is called the training set and is used during the learning phase of the model, while the second part is called the test set and is used to determine the ability of the model to associate an unknown pattern with some pattern that has been previously learned. The Amαβ, like other supervised methods, has three phases: Learning phase, recovery phase, and test phase. The use of the training set or the test set depends on the phase.

#### 2.6.1. Learning Phase

The purpose of this phase is to build the associative memory from the patterns of the training set The techniques for encoding the patterns in a learning matrix M depend on the mathematical model of each type of memory.

**Figure 3:**
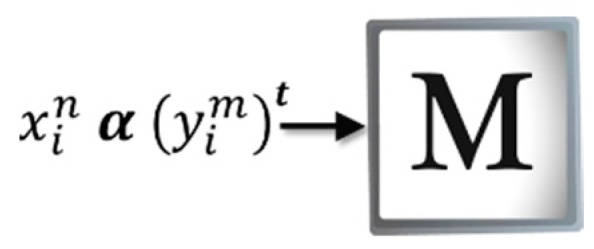
Learning phase of the Amαβ.

Figure 3 shows how ***M*** is created in the Amαβ, which is constructed from each of the input patterns that belong to the training set The patterns must be binary vectors denoted by $x_{i}^{n}|\forall n\in \left\{1,2,\ldots ,p\right\},\;\forall i\in \left\{1,2,\ldots ,l\right\}$, where p = number of input patterns and l = pattern size. It should be noted that the vector $x_{i}^{n}$ is formed from the representative features of the object to be recognized. Therefore, the vector $y_{c}^{m}$ is also binary and is defined as $y_{i}^{m}|\forall m\in \left\{1,2,\ldots ,c\right\},\;\forall i\in \left\{1,2,\ldots ,l\right\}$, where c = number of classes and l = pattern size. Thus, for each input pattern, $x_{i}^{n}$ is associated with a desired output $y_{i}^{m}$, which results in the ordered pair ($x_{i}^{n},y_{i}^{m}$).

(5) $$x_{i}^{n}=\left[\begin{array}{c} \begin{array}{c} \begin{array}{c} x_{1}^{n}\\ x_{2}^{n} \end{array}\\ . \end{array}\\ .\\ .\\ x_{l}^{n} \end{array}\right]\;\;\;\;\;\;\;\;y_{i}^{m}=\left[\begin{array}{c} \begin{array}{c} \begin{array}{c} y_{1}^{m}\\ y_{2}^{m} \end{array}\\ . \end{array}\\ .\\ .\\ y_{l}^{m} \end{array}\right]$$

To build M the following steps are followed:

(a) The ordered pair ($x_{i}^{n},y_{i}^{m}$) is taken and the operator α defined by Table 1 is applied. Therefore, for each ($x_{i}^{n},y_{i}^{m}$) a primary matrix is generated which is defined as ${\left[M_{ij}^{\prime }\right]}^{n}|\forall n\in \left\{1,2,\ldots ,p\right\},\forall i,j\in \left\{1,2,\ldots ,l\right\}$.
  (6) $${\left[M^{\prime }\right]}^{n}={\left[x^{n}\;\alpha \;{\left(y^{m}\right)}^{t}\right]}_{lxl}=\left[\begin{array}{c} \begin{array}{c} \begin{array}{c} x_{1}^{n}\\ x_{2}^{n} \end{array}\\ . \end{array}\\ .\\ .\\ x_{l}^{n} \end{array}\right]\alpha \left[y_{1}^{m}\;\;y_{2}^{m}\;\;\ldots \;\;y_{l}^{m}\right]$$
(b) The max operator ⋁ is applied to determine the maximum value of each cell α ($x_{i}^{n},y_{i}^{m}$) of the matrices ${\left[M^{\prime }\right]}^{n}$ generated in step a.
  (7) $$v_{ij}=\overset{n=1}{\underset{p}{⋁}}{\left[\alpha {\left(x_{i}^{n},(y_{i}^{m}\right)}^{t})\right]}^{n}$$

The result obtained is a learning matrix M.

(8) $$M=\left[\begin{array}{cccc} v_{11} & v_{12} & \cdots & v_{1l}\\ v_{21} & v_{22} & \cdots & v_{2l}\\ . & . & \cdots & .\\ . & . & \cdots & .\\ . & . & \cdots & .\\ v_{l1} & v_{l2} & \cdots & v_{ll} \end{array}\right]$$

It should be considered that the Amαβ is able to function in two self-associative and heteroassociative modes, the difference between the two lies in the definition of the vector $y_{i}^{m}$, thus that, in a hetero-associative memory αβ, it is expected that the pattern $x_{i}^{n}$ has an assigned class. Therefore, $x_{i}^{n}\neq y_{i}^{m}$ since $x_{i}^{n}$ represents the features of an object while $y_{i}^{m}$ represents the class to which the object belongs. In the Amαβ that is self-associative $x_{i}^{n}=y_{i}^{m}$, which means that the memory tries to associate the features of the object that they want to classify with the features of the objects that Amαβ has previously learned, thus that, in the self-associative memories, p=c.

#### 2.6.2. Recovery Phase

**Figure 4:**
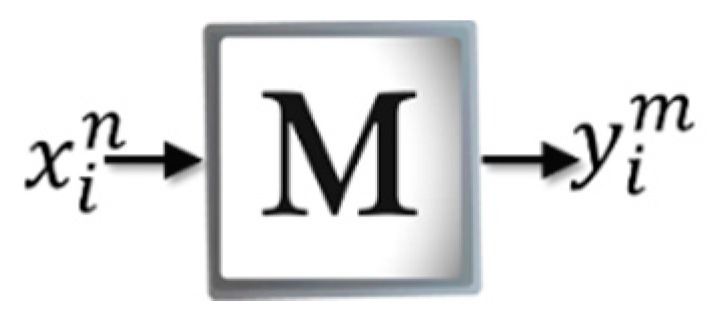
Recovery phase of the Amαβ.

Once M has been constructed, the efficiency of Amαβ in retrieving or recalling previously learned patterns is determined. Figure 4 shows a schematic of the recovery phase where each of the patterns $x_{i}^{n}$ of the training set is presented to matrix M. During this phase, the performance of the Amαβ is evaluated to associate the pattern $x_{i}^{n}$ with some of the patterns that it has previously learned, thus at the exit a binary vector represented by $y_{i}^{m}$ is obtained. Thus, if it is a heteroassociative memory, the expected output $y_{i}^{m}$ will be equal to the associated class of the corresponding pattern. However, if it is a self-associative memory then it is expected that $y_{i}^{m}$ = $x_{i}^{n}$.

The steps that are followed during the recovery phase are:

(c) The patterns $x_{i}^{n}$ are presented to M, and the operator β shown in Table 1 is applied, which results in n matrices such as the one shown below.
  (9) $$y_{i}^{m}=\left[\begin{array}{cccc} \beta \left(v_{11},x_{1}^{n}\right) & \beta \left(v_{12},x_{1}^{n}\right) & \cdots & \beta \left(v_{1l},x_{1}^{n}\right)\\ \beta \left(v_{21},x_{2}^{n}\right) & \beta \left(v_{12},x_{2}^{n}\right) & \cdots & \beta \left(v_{2l},x_{2}^{n}\right)\\ . & . & \cdots & .\\ . & . & \cdots & .\\ . & . & \cdots & .\\ \beta \left(v_{l1},x_{l}^{n}\right) & \beta \left(v_{l2},x_{l}^{n}\right) & \cdots & \beta \left(v_{ll},x_{l}^{n}\right) \end{array}\right]$$
(d) Finally, to obtain the output vector $y_{i}^{m}$, it is necessary to apply the min operator ⋀ on each row of the matrix obtained in step c.
  (10) $$y_{i}^{m}=\left[\begin{array}{c} \overset{l}{\underset{i=1}{⋀}}\beta \left(v_{1i},x_{i}^{n}\right)\\ \overset{l}{\underset{i=1}{⋀}}\beta \left(v_{2i},x_{i}^{n}\right)\\ .\\ .\\ .\\ \overset{l}{\underset{i=1}{⋀}}\beta \left(v_{li},x_{i}^{n}\right) \end{array}\right]$$

Steps a–d can be summarized under the expression

(11) $$y_{i}^{m}=\overset{l}{\underset{i=1}{⋀}}\beta \left\{\left[\overset{p}{\underset{n=1}{⋁}}{\left[\alpha {\left(x_{i}^{n},(y_{i}^{m}\right)}^{t})\right]}^{n}\right],x_{i}^{n}\right\}$$

#### 2.6.3. Test Phase

In this phase, the aim is to determine the performance of Amαβ when classifying patterns that have not been learned by memory.

**Figure 5:**
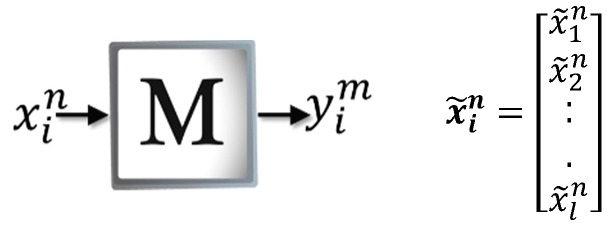
Test phase of the Amαβ.

The test phase is shown in Figure 5, where the patterns that do not belong to the training set and therefore contain subtractive noise are presented to matrix M. These patterns are denoted by ${\tilde{x}}_{i}^{n}$ and are stored in a database called a test set The test phase is very similar to the recovery phase, since the same steps c and d are followed, which are summarized in Equation (13). However, the patterns that are presented to the learning matrix M are those that are stored in the test set

(12) $$y_{i}^{m}=\overset{l}{\underset{i=1}{⋀}}\beta \left\{\left[\begin{array}{cccc} v_{11} & v_{12} & \cdots & v_{1l}\\ v_{21} & v_{22} & \cdots & v_{2l}\\ . & . & \cdots & .\\ . & . & \cdots & .\\ . & . & \cdots & .\\ v_{l1} & v_{l2} & \cdots & v_{ll} \end{array}\right],\left[\begin{array}{c} \begin{array}{c} \begin{array}{c} {\tilde{x}}_{1}^{n}\\ {\tilde{x}}_{2}^{n} \end{array}\\ . \end{array}\\ .\\ .\\ {\tilde{x}}_{l}^{n} \end{array}\right]\right\}$$

### 2.7. Using CMKNN

It is important to note that during the recovery and test phases of the Amαβ an associated output pattern of the same size of the input pattern is obtained, therefore p=c, which only in the recovery phase is fulfilled $x_{i}^{n}=y_{i}^{m}$, therefore, for the test phase, it is necessary to establish consistent relationship measures between the output pattern associated with the memory and the patterns that belong to the training set to finally make the assignment of the class. Therefore, it is proposed to adapt the approach presented by Zhu etal. [49] and Zhang et al. [50]. The basic idea is to use a correlation matrix (CM) to establish a measure of relationship between the two patterns, in the original work it is proposed to use Frobenius matrix norm to obtain the correlation coefficient which is used to determine the K value for the KNN algorithm (K-Nearest Neighbors), however, one of the adjustments is to use Pearson Correlation Matrix [51,52], thus that in this work the Pearson Correlation Coefficient (PCC) is proposed to replace the Euclidean distance that is typically used with KNN.

Once the associated output pattern of the Amαβ is obtained in its binary form (see Algorithm 1), it is proposed to return it to its decimal form and begin its analysis to relate it to the patterns of the training set and assign it a class. The training set is represented as X and each pattern is $x_{n,i}\;|\forall n\in \left\{1,2,\ldots ,p\right\},\;\forall i\in \left\{1,2,\ldots ,l\right\}$ where p is the number of input patterns and l is the number of features of each pattern, while $y_{m,i}$ represents the output pattern associated with the Amαβ in decimal form.

(13) $$X=\left[\begin{array}{cccc} x_{11} & x_{12} & \cdots & x_{1l}\\ x_{21} & x_{22} & \ldots & x_{2l}\\ . & . & \cdots & .\\ . & . & \cdots & .\\ . & . & \cdots & .\\ x_{p1} & x_{p2} & \cdots & x_{pl} \end{array}\right]$$

To obtain the Pearson Correlation Factor, Equation (8) is applied between the associated pattern $y_{m,i}$ and each of the patterns of the training set $x_{n,i}$.

(14) $$r_{xm}=\frac{\sum_{i=1}^{l}\left(x_{n,i}-\;{\bar{x}}_{n,i}\right)\left(y_{m,i}-{\bar{y}}_{m,i}\right)}{\sqrt{\sum_{i=1}^{l}{\left(x_{n,i}-\;{\bar{x}}_{n,i}\right)}^{2}\sum_{i=1}^{l}{\left(y_{m,i}-{\bar{y}}_{m,i}\right)}^{2}}}$$

The result obtained is a correlation matrix X.

(15) $$X=\left[\begin{array}{cccc} 1 & \cdot \cdot & \cdots & \cdot \cdot \\ r_{2m} & 1 & \cdots & \cdot \cdot \\ r_{3m} & r_{32} & \cdots & \cdot \\ \cdot & \cdot & \cdots & \cdot \\ \cdot & \cdot & \cdots & \cdot \\ r_{pm} & r_{p2} & \cdots & 1 \end{array}\right]$$

The first column provides information about the relationship between $y_{m,i}$ and each of the patterns of the training set, thus KNN is applied to determine the greatest association and the corresponding class assignment.

(16) $$C=arg\;\underset{c}{\max r}\left(y_{m},x_{n}\right)$$

## 3. Experiments and Results

### 3.1. Database Description

To test the performance of the proposed hybrid model Amαβ-CMKNN three databases were included which are integrated with faces under different facial expressions, besides two of them, we also tested the model under different poses and real environmental conditions. The databases are described in detail below:

#### 3.1.1. The Extended Cohn-Kanade Dataset (CK+) 

**Figure 6:**
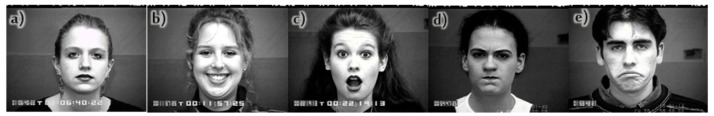
Cohn-Kanade plus database, (**a**) neutral, (**b**) happiness, (**c**) surprise, (**d**) anger, (**e**) sadness.

The CK+ database originally included 593 sequences from 123 subjects between 18 to 50 years of age. 69% were women, 81% Euro-American, 13% African-American and 6% other groups. The sequences begin in a neutral pose and end when the facial expression has reached the cusp. The study subjects perform six facial expressions (see Figure 6), one in each sequence, which are analyzed with FACS action units that describe the subject´s expression in terms of units of action (UA). The image sequences are digitized with a resolution of 640 × 480 or 640 × 490 pixels with an 8-bit precision or 24-bit grayscale. Images are available in PNG and JPEG format.

In this paper, we considered 64 subjects from the 123 contained in the CK+ database. The sequences were selected to maintain uniformity in the number of samples per person. For this reason, 30 images of each person were selected, including 6 images of each of the 5 facial expressions (neutral, happiness, surprise, anger and sadness) used for the present study where the face shows the maximum level of expression. Thus, from this database, 1920 images were considered.

#### 3.1.2. The CAS-PEAL-R1 Database

Another database that was used in this paper is the CAS-PEAL-R1 face database collected under the sponsor of the Chinese National Hi-Tech Program and ISVISION Tech. Co. Ltd. [53], the database is integrated of 30,900 images captured from 1040 subjects of study. The images were captured under different conditions of lighting, facial expressions, and pose. Among the characteristics of interest for this research are: 379 subjects have images with five different expressions (open mouth, frown, close eyes, smile, and surprise), in addition to including images in which the study subjects present different controlled poses in neutral facial expression.

**Figure 7:**
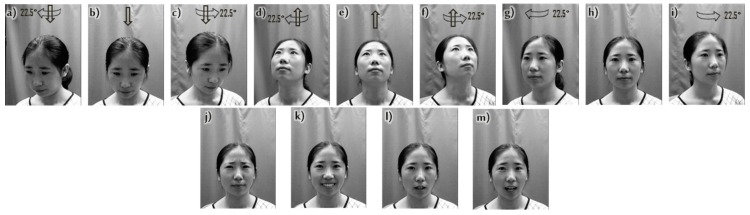
Sample of the CAS-PEAL-R1 database, (**a**) down and rotation to the right, (**b**) down, (**c**) down and rotation to the left, (**d**) up and rotation to the right, (**e**) up, (**f**) up and rotation to the left, (**g**) rotation to the right, (**h**) frontal, (**i**) rotation to the left, (**j**) frown, (**k**) smile, (**l**) open mouth, (**m**) surprise.

For the purposes of this work, it was considered to take for each of the 1040 subjects included in CAS-PEAL-R1 the images corresponding to four facial expressions (frown, smile, open mouth, and surprise), as well as nine images in different poses under neutral expression, which gives a total of 13 images per subject and therefore 13,520 total images (see Figure 7).

#### 3.1.3. Own Database Face-MECS

Finally, for this research, an own database was created with 270 videos of faces from 54 people with an approximate duration of 3 seconds per video, each individual was instructed to pose under 5 facial expressions including happiness, surprise, anger, sadness, and neutral, therefore, a total of 24,300 static images were generated considering that the Logitech C310 camera captures 30 frames per second. The samples contain different pose angles that were not controlled because they were captured when using the proposed recognition system in outdoor and indoor environments (see Section 4), thus the type of lighting was uncontrolled.

**Figure 8:**
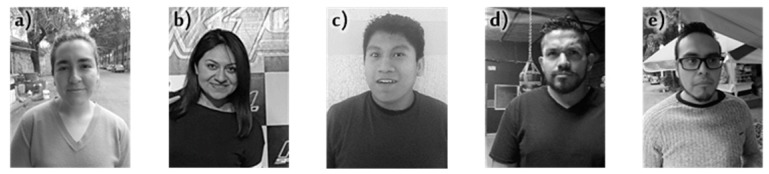
Examples of frames of the Face-MECS database, (**a**) neutral, (**b**) happiness, (**c**) surprise, (**d**) anger, (**e**) sadness.

For the experiments, a total of 24,300 frames included in the Phase-MECS database were considered, the difference is that they are processing is done in the video at 30 fps. Figure 8 shows examples of frames extracted from the videos with which the tests described in Section 3.3 and Section 3.4 were performed. In Section 3.3, the database was used to evaluate, under real conditions, the behavior of the proposed HLF and AAM models in the detection and extraction of facial characteristics, while in Section 3.4 the performance of the proposed hybrid model against other models framed in artificial intelligence is evaluated.

### 3.2. Selection of Characteristic Features

Within the present work two types of significant characteristics were proposed to describe the face in an interpretation of quantitative parameters, since during the study we presupposed that the correct selection of them will have a positive effect on the performance of pattern recognition models, so it is fundamental key to determine the number and type of features that the algorithm must learn, as well as the alterations suffered by the features when there are different angles of the face in relation to the camera.

#### 3.2.1. Experiment 1

**Figure 9:**
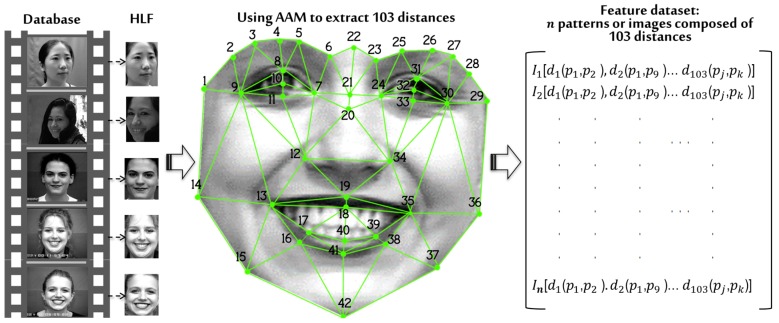
Dataset generated from the mesh resulting from the application of the active appearance model (AAM), where 42 points are located, from which 103 distances are generated for each processed face.

Figure 9 shows the proposed 42 points, which are strategically distributed, framing the most important elements of the face such as nose, eyes, and mouth. To form a mesh, the points are considered nodes joined with other adjacent nodes forming triangles that do not overlap. The AAM technique described in Section 2.3 was used to detect the 42 points proposed and to establish the relationship between them.

As a result, the ordered pair $p_{j}\left(x,y\right)$ is obtained, which indicates the position of a given point within the image. Then, the distances of the sides of each triangle formed are determined. Therefore, a total of 103 distances are obtained by each of the patterns or faces included in the three proposed databases represented as *n* in Figure 9. Ttherefore for the CK+ database with n = 1920 patterns, for CAS-PEAL-R1 n = 13,520 patterns and finally for Face-MECS n = 24,300 patterns, where each pattern is formed with 103 features that characterize each pattern or face. While to determine the length of the straight line joining two points the following formula was used:

(17) $$d\left(p_{j},p_{k}\right)=\sqrt[{2}]{\left(x_{j}-x_{k}\right)+\left(y_{j}-y_{k}\right)}$$

#### 3.2.2. Experiment 2

**Figure 10:**
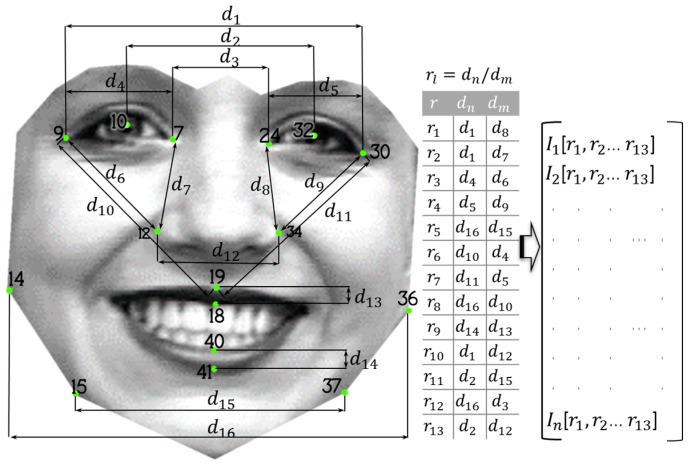
Ratios between face distances considered for Experiment 2.

For this experiment 16 of the 42 original points are selected. Another fundamental change is that no triangles are formed since for this experiment ratios are established between distances that are considered little affected at the moment of performing a facial expression but at the same time allow differences between the faces to be identified. The 16 distances denoted with d in Figure 10 are used to generate 13 ratios that are converted into the features of each face, thus, obtaining three new databases with vectors generated from the images stored in the databases of CK+, CAS-PEAL-R1, and Face-MECS, where each vector becomes a pattern composed of 13 features that parameterize the face in relation to its proportions.

In Figure 2 it can be seen that steps 1 and 2 are dedicated to the extraction of features. However, step 3 aims to implement a pattern recognition model with the ability to learn the features that have been obtained in the previous steps. In this sense, it is proposed to use Amαβ-CMKNN as a classifier. However, the Amαβ is binary, thus it is necessary to perform a conversion of the features obtained for each pattern, since they are in decimal form. Because the Amαβ has its principles in mathematical morphology they perform better when learning defined forms.

Therefore, to convert from decimal to binary digits, the present study makes use of the Johnson Möbius code [54] instead of the classic binary code. For this purpose, the steps described in Algorithm 1 are followed. Each of the strings generated by using the Johnson Möbius code can be defined as $x_{i}^{n}|\forall n\in \left\{1,2,\ldots ,p\right\},\;\forall i\in \left\{1,2,\ldots ,l\right\}$ where p = number of patterns or images, while l = is the dimension of the concatenated string of each pattern.


**Algorithm 1. Convert a Johnson Möbius Code**
**Input**: $I_{m}$ images with $f_{1}\left(p_{1},p_{2}\right),f_{2}\left(p_{1},p_{9}\right),\ldots ,f_{103}\left(p_{j},p_{k}\right)$. **Output**: Database in Johnson Möbius code.     1.- **for**
m∈1…M|M=1920
**do** //Pattern iteration     2.-    **for**
f∈1…F|F=103 or 13 **do** // Features iteration     3.-    Multiply $I_{m}\left[f_{r}\right]*100$ //The values of the features are converted to integers.     4.-    Convert the integer value of each feature $I_{m}\left[f_{r}\right]$ into ones [51].     5.-    Normalize the length of each feature by adding zeros to the left of the string of ones, taking as reference the largest value of the column of each feature.     6.-    **end for**     7.-     Concatenate the strings obtained from each feature in an orderly manner, forming a binary string for each pattern.     8.- **end for**

At this point, it is necessary to apply the k-fold cross validation method with ***k*** = 10 to generate the training set and test. For this, the database is randomly divided into k subsets of approximately the same size, where the subsets ***k*** − 1 constitute the training set and the remaining subset forms the test set Therefore, for the CK+ database of the 1920 patterns that make up the original database, 1728 patterns constitute the training set, while the test set is composed of the remaining 10%, i.e., 192 patterns. The patterns used in each of the 10 rounds for each proposed database are shown in Table 2. It is necessary to repeat the cross-validation process ***k*** times. For this, 10 rounds are made by making a rotation of the ***k*** subset that generates the test set. The goal is for each pattern to be part of the test set in one of the rounds, allowing the performance of Amαβ-CMKNN to be tested with different patterns in the learning and test sets. It should be remembered that at this point the patterns of both the training set and the test set are binary strings of 0 s and 1 s. Therefore, it is possible to begin the learning phase of Amαβ in its self-associative mode where $x_{i}^{n}$ = $y_{i}^{m}$, generating the learning matrix with the 1728 training set patterns for the CK+ database, the procedure for which is described in Section 2.6—Phase of Learning, where steps a and b are followed.

Once the learning matrix M is obtained, the ability of the algorithm to remember each of the learned patterns is determined, following steps c and d of Section 2.6—Recovery Phase. The patterns used in this phase are the same as those with which matrix M was trained because in this phase the aim is to evaluate the capacity of the Amαβ-CMKNN to recover the previously learned patterns. Finally, in the test phase, the same steps as those followed during the recovery phase are applied, but with the difference being that the patterns presented to M are those belonging to the test set, i.e., the 192 different patterns in each of the 10 rounds of the k-fold cross validation denoted as ${\tilde{x}}_{i}^{n}$.

### 3.3. Results of the Detection Phase of the Face and Characteristic Points

**Figure 11:**
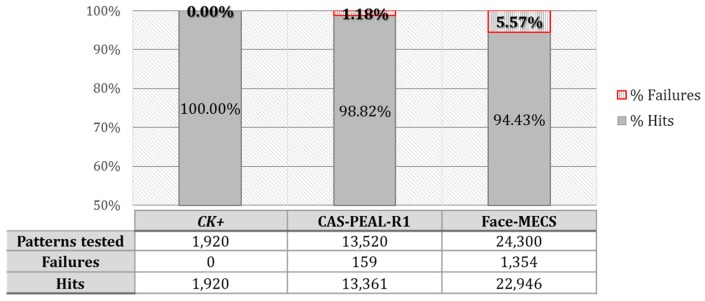
Results obtained by applying the Haar-like features (HLF) in the proposed databases.

During this stage the performance of the HLF algorithm used to detect faces was evaluated (see Section 2.2) and the AAM used for the extraction of characteristics (see Section 2.3), to evaluate the performance of HLF and AAM the databases CK+, CAS-PEAL-R2 and Face-MECS were used. The results of the tests performed on the three databases using HLF can be seen in Figure 11, where, when using the Face-MECS database, the HLF algorithm has the highest error rate of 5.57%, while using CK+ all the faces were detected.

Based on the results obtained from face detection and segmentation tests, the next step is to determine the performance of the AAM model. For this, only the images were taken in, which the HLF algorithm found a positive match of the face within an image or frame as the case may be, thus that to determine the efficiency of the AAM model during the test. The three databases were used considering that for CK+ a total of 1920 faces were detected, for CAS-PEAL-R1 AAM was tested on 13,361 faces detected and finally for Face-MECS a total of 22,946 faces detected, were evaluated (see Figure 11).

**Figure 12:**
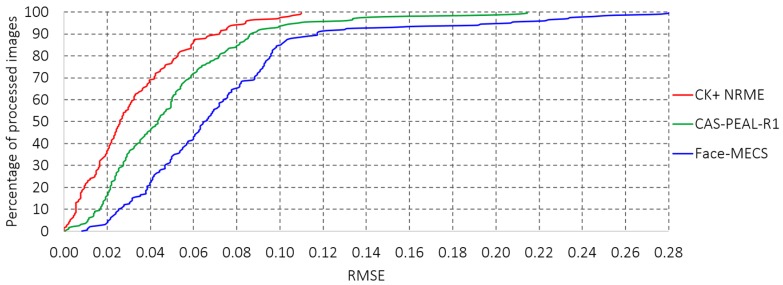
Root-Mean-Squared Error obtained by applying the AAM in the proposed databases.

The results of the evaluation of the AAM in the databases are shown in relation to the location error factor $e_{i}^{k}$, which is calculated from the difference between the expected position and the one generated by the AAM of each landmark, where k is the number of landmarks to be evaluated; for this test *k* = 42 landmarks proposed in Experiment 1 (see Section 3.2.1), it is necessary to say that for Experiment 2 a selection of 16 points of the 42 landmarks of Experiment 1 is made, thus to evaluate the performance of AAM is considered to assess the maximum of landmarks (42 points). Once the error of each landmark point-to-point $e_{i}^{k}$ is determined, the next step is to calculate the Root-Mean-Squared Error (RMSE) for each of the faces, which allows us to measure how much error there is between two data sets, the results obtained from the RMSE are shown in Figure 12. The results obtained from the RMSE are shown in reference [40], it is possible to say that $e_{i}^{k}$ < 1.0 can be taken as an acceptable error criterion under a controlled environment.

### 3.4. Performance Evaluation Hybrid Model Amαβ-CMKNN

For this part of the process the programming of each of the algorithms was done in C++, using the Microsoft Visual Studio development environment, installed on a laptop with an Intel Core i7 processor, 24 GB of RAM, Geforce GTX 980 graphics card, and running the Microsoft Windows 10 operating system. For the implementation of the Haar-Like Feature (HLF) technique used in face identification and the AAM applied in the detection of characteristic points, the open source computer vision OpenCV 2.4 libraries were used. However, for the implementation of the Amαβ-CMKNN, it was necessary to program the three phases of the memories and to create a library of its own.

To test the Amαβ-CMKNN, two experiments were conducted, as described in Section 3.2.1 and Section 3.2.2, Experiments 1 and 2 were proposed to determine how the selection of facial characteristics influences the performance of the proposed hybrid model, this during the recovery and test phases, for this purpose the databases CK+, CAS-PEAL-R1, and Face-MECS have been used, which test the Amαβ-CMKNN with faces of subjects of both sexes, different races, facial expressions, poses and environmental conditions. The k-Fold Cross Validation method with ***k*** = 10, was used to form the training set in the k-rounds with 90% of the faces of the subjects in the database and the test set with the remaining 10%. The goal is to integrate the training and test sets thus that the test patterns rotate in each of the 10 rounds, in this way during the rotation of all the faces will have been at some point in the test set Now, two experiments were proposed in which the fundamental difference lies in the number of features considered to define each person, in Experiment 1 we obtained 103 distances generated from 42 points that frame the main elements of the face, while in Experiment 2 the aim was to reduce the number of features by selecting those that are able to characterize a face while being less affected by changes of facial expression. 16 points were selected carefully from the 42 original points, and then from these 16 points, 13 proportions were formed considering the distances between the points.

The tests applied to the proposed hybrid model was designed to determine their performance both in the process of remembering the learned patterns and in the classification of new patterns, which can be divided into the recovery phase and the test phase.

#### 3.4.1. Recovery Phase Results

During the recovery phase, the aim is to determine the accuracy of the proposed model when trying to remember the faces that you have previously learned, therefore during this phase in each round new learning and test sets are formed by rotating the patterns of the original database containing the characteristic binary vectors extracted from the faces of study subjects contained in each of the proposed databases. In that sense, in each round, the training and test sets are generated in order to rotate the patterns that belong to each set, which results in 10 different learning matrices M. Therefore, one should evaluate the recall ability of each M. For this, it is necessary that in each round, one by one, the patterns $x_{i}^{n}$ of the learning set are presented to the matrix M previously trained, where the operator β and max ⋁ are used to generate an associated pattern $y_{i}^{m}$. Finally CMKNN is used for the assignment of the class, and after finishing with all the patterns of the training set, the percentage of the obtained successes was calculated (see Section 2.6—Recovery phase). It is important to remember that in the recovery phase only the training set is used, since the objective is to determine the performance of the Amαβ-CMKNN when remembering the patterns learned.

**Figure 13:**
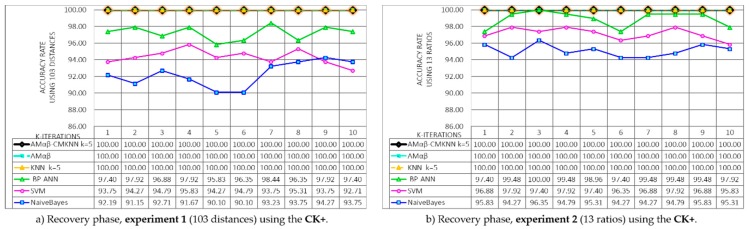

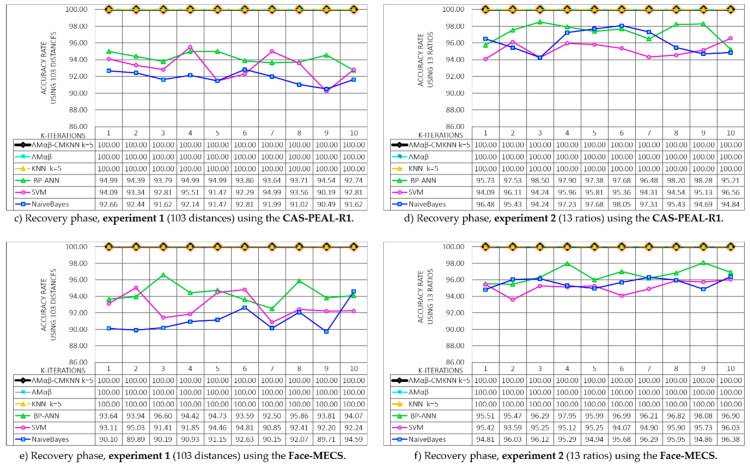
Results of the 10 test rounds of the k-fold-cross validation method applied during the recovery phase of Experiments 1 and 2 using the CK+, CAS-PEAL-R1 and Face-MECS databases.

Since two experiments and three databases are proposed, the results of the model Amαβ-CMKNN during the recovery phase have been divided into six graphs shown in Figure 13, additionally, it is equally important to determine the competitiveness of the proposed model compared to other models widely used in face recognition literature. The behavior of other models was determined using the same databases, the graphs also include the results obtained during the recovery phase of the models: The original associative memories αβ (MAαβ) model, nearest K-neighbors (KNN) [55,56], Bayesian Network [57], Support Vector Machines (SVM) [55] and Neural Network Backpropagation (BP-ANN) [58].

In Figure 13a,c,e shows the results of the recovery phase obtained in each of the 10 iterations of the k-fold cross validation using 103 distances of each face (Experiment 1), each graphic belongs to the databases CK+, CAS-PEAL-R1 and Face-MECS, the results show that the original model of Amαβ, the KNN model with centroid and k = 5, and the proposed hybrid model Amαβ-CMKNN, managed to remember 100% of all the faces (training set) learned in the 10 rounds. Whereas, for CK+ the average obtained from BP-ANN = 97.24, SVM = 94.32 and NaivesBayes = 92.29; for the database CAS-PEAL-R1 the average of the models was BP-ANN = 94.16, SVM = 93.11 and NaivesBayes = 91.83, and finally in the Face-MECS database the average obtained was BP-ANN = 92.84, SVM = 92.84 and NaivesBayes = 91.14.

For the recovery phase, the models were also tested using 13 ratios to parameterize the face (Experiment 2). The results are shown in Figure 13b,d,f, where the performance of the proposed hybrid model and other state of the art models are tested in the three proposed databases. Again the Amαβ, KNN and Amαβ-CMKNN models recalled the entire set of training learned in the 10 rounds of the recovery phase, additionally, it is possible to observe that in a general way all the models in each database present better performances with the 13 ratios taken from the face than with the 103 distances of Experiment 1.

#### 3.4.2. Test Phase Results

Another evaluation that was performed on the Amαβ-CMKNN is the one that corresponds to the test phase. The aim of this evaluation was to determine the performance of the model when trying to relate a pattern that the memory has not learned with some of the patterns that it learned during its learning phase, thus that, during this evaluation, the memory functions as a classifier. The Amαβ-CMKNN algorithm was tested 10 times in each of the two experiments, where the training and test sets were generated using k-fold cross validation, and in which the patterns that integrate the sets were rotated in each round thus that each pattern in one of the rounds forms part of the test set once. Additionally, to determine the competitiveness of Amαβ-CMKNN it was necessary to compare the results obtained with the performance of other classifiers commonly used in the problem of facial recognition, therefore, the original model of the Amαβ, Naive Bayes (NB), Euclidean distance with centroid, and KNN, Support Vector Machines (SVM), and Backpropagation Artificial Neural Networks (BP-ANN) were the proposed models to compare the performance of the Amαβ-CMKNN.

For the test phase, it is necessary to divide the selected database into two parts, the training set and the test set, this segmentation is done in 10 rounds in which the patterns extracted from the faces are rotated. Therefore, for the CK+ database in which all the faces were detected with HLF (see Figure 11) the training set in each round has a size of 1728 patterns that characterize each face and the rest of the faces detected make up the test set which has a size of 192 patterns. The database CAS-PEAL-R1 has a total of 13,520 faces of which 13,361 were detected, thus that the size of the test sets in each round is 12,025 patterns and the training sets have a size of 1336. For the Face-MECS database that integrates 24,300 faces, 22,946 faces were successfully located during the detection step, thus, the size of the training sets was 20,652 and the test sizes of 2294 patterns.

**Figure 14:**
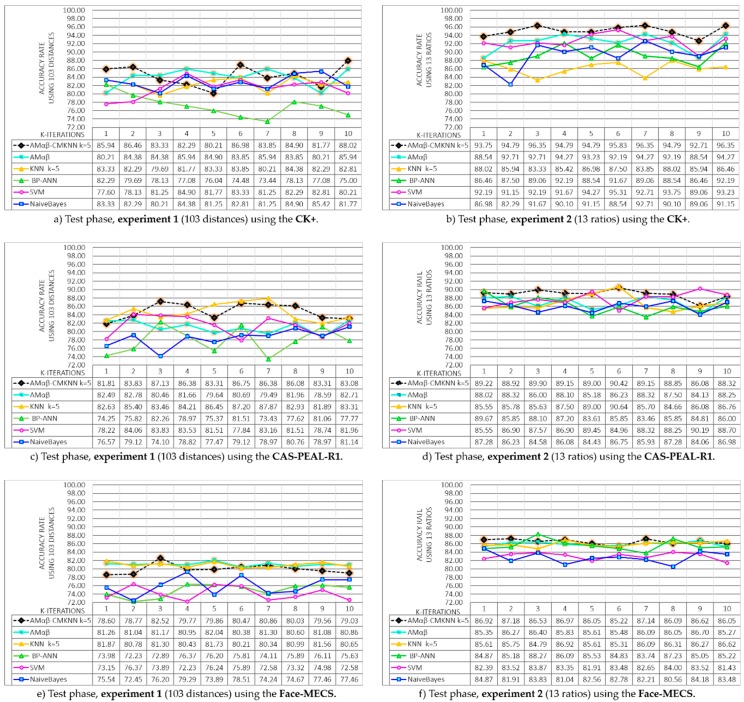
Results of the 10 test rounds of the k-fold-cross validation method applied during the test phase of Experiments 1 and 2 using the CK+, CAS-PEAL-R1 and Face-MECS databases.

Figure 14 shows the results obtained during the test phase separated by experiments and type of database used during the test, where it is possible to observe that the performance of all the models present a better overall performance when tested with the characteristics of Experiment 2, being the CK + database where better results are obtained, however, with the Face-MECS own database the results obtained are the most real when working the system in real conditions, the proposed Amαβ-CMKNN model demonstrates its competitiveness in all the graphs presented in the Figure 14, but in the graph f) is where it is observed that the models that compete strongly are the original model of the Amαβ and the neural network.

It is important to say that for each round a M learning matrix is created from the training sets and with the test set the performance of the proposed model and that of the models that frame the state of the art are tested. Thus to try the Amαβ-CMKNN in each round, the patterns ${\tilde{x}}_{i}^{n}$ of the test set were presented one by one to the matrix M, where the operators β and max ⋁ were used to obtain an output pattern $y_{i}^{m}$. This pattern was used to indicate with which pattern of the previously learned the memory had found the greatest similarity using CMKNN. Afterwards, for each pattern of the test set, it was determined if the classification was correct, and at the conclusion of the n patterns (test set), the percentage of successes for each round was calculated (see Section 2.6—Test phase).

**Figure 15:**
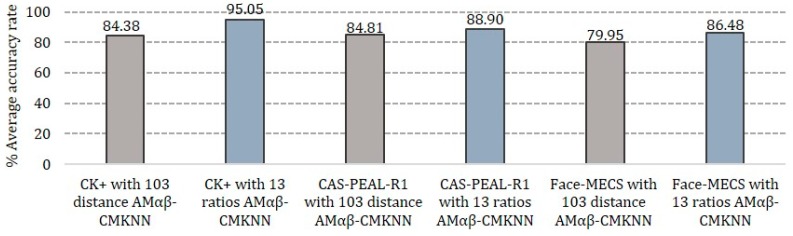
Results of Experiments 1 and 2 obtained in the test phase.

In Figure 15, the results of the performance of the Amαβ-CMKNN under the two experiments conducted for the test phase are shown, while in gray the results of the average of the 10 rounds obtained from Experiment 1 are shown in each proposed database, also the results of Experiment 2 are shown in blue where it is possible to observe that in general the performance of the Amαβ-CMKNN it is better in Experiment 2, in which proportions of the human face are considered as characteristics to form the pattern of each face and, therefore, the parameters are less sensitive when the faces have different angles or under some gesticulation, since the distances taken in Experiment 1 are subject to change while turning the face with respect to the camera or the face is deformed in some facial expression.

It is also possible to observe that the database in which the proposed hybrid model has lower assertiveness rates is in Face-MECS with a performance of 86.48%, this is because this database has frames under uncontrolled conditions.

**Figure 16:**
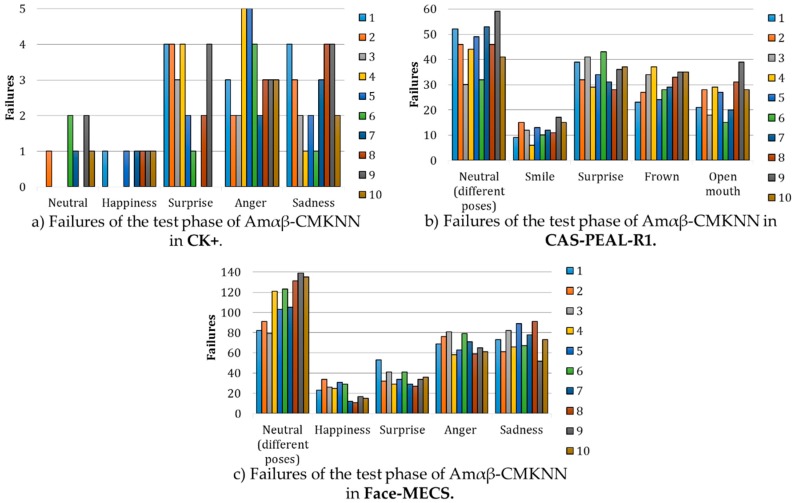
Failures obtained during the test phase of Amαβ-CMKNN analyzed by each facial expression and database.

Figure 16 shows the behavior of the Amαβ-CMKNN during the recognition of faces under different facial expressions using only 13 ratios of the face, each graph shows the faults cataloged by facial expression depending on the analyzed database, in each expression the results of failures obtained in each of the 10 rounds of the k-fold cross validation during the test phase are shown. In Figure 16a–c it is possible to observe that regardless of the database in which the model was tested, where fewer errors were obtained is when the face expresses happiness, whose results agree with what was reported in reference [59] where it is concluded that the way of smiling of each person is particular thus it can be used as a significant feature to recognize the subject of study.

It can also be seen that in Figure 16b,c the errors in the expression in neutral increase with respect to the Figure 16a, nevertheless, it is important to consider that in the databases CAS-PEAL-R1 and Face-MECS include in their expression neutral different angles of the face with respect to the camera.

## 4. Implementation and System Integration

**Figure 17:**
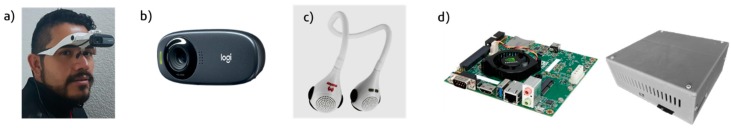
Devices that integrate the face recognition system for people with disabilities. (**a**) Headband-shaped structure, (**b**) webcam Logitech c310, (**c**) sports speakers brand master model MS-SPORTSPK, (**d**) NVIDIA Jetson TK1 developer kit with 3D printed case.

Once the competitiveness of the proposed model in the task of face recognition under different facial expressions has been demonstrated, the next objective is to carry out the processes of implementing the devices and adapting them to people with disabilities and the integration of the system identified in Figure 1b,c. For this purpose, the use of four devices is proposed, the first is a structure that can be mounted easily in the head (see Figure 17a), the attachment is designed as a headband thus that it does not interfere with the visual field of the eyes, because although the system is designed for people with a visual disability it can also be used by people suffering from prosopagnosia, which is a cognitive disorder characterized by the inability to recognize faces, thus in these cases it is important to keep the visual channel clear. The second device is a webcam c310 Logitech brand whose maximum resolution is 720 ppp/30 fps (see Figure 17b), the camera is mounted in the form of a Cyclops in the structure. The third device is a flexible sports speaker with Bluetooth connection and a battery with a maximum duration of 2.5 h, which are placed around the neck (see Figure 17c). The latest device is an NVIDIA Jetson TK1 mini PC which has an NVIDIA Kepler GPU with 192 CUDA Cores and NVIDIA 4-Plus-1™ Quad-Core ARM® Cortex™-A15 CPU (see Figure 17d).

For the integration of the system it was necessary to migrate the proposed recognition algorithms to the Jetson TK1, since it should be considered that this PC works with an operating system Ubuntu 14.04LTS, likewise adjustments were made so that each algorithm works in video and real time, for this, own libraries were created in addition to using the set of libraries integrated in OpenCV 3.2.0.

Once the algorithms run correctly using the Logitech webcam, a database was made with faces of people that the individual wanted to recognize, the capture of the images was done under different conditions of lighting, facial expression, and pose. The own database is integrated of 325 images generated from 25 subjects with 13 samples per person, later the images are processed to extract the characteristic vectors which are learned by the proposed hybrid model Amαβ-CMKNN.

**Figure 18:**
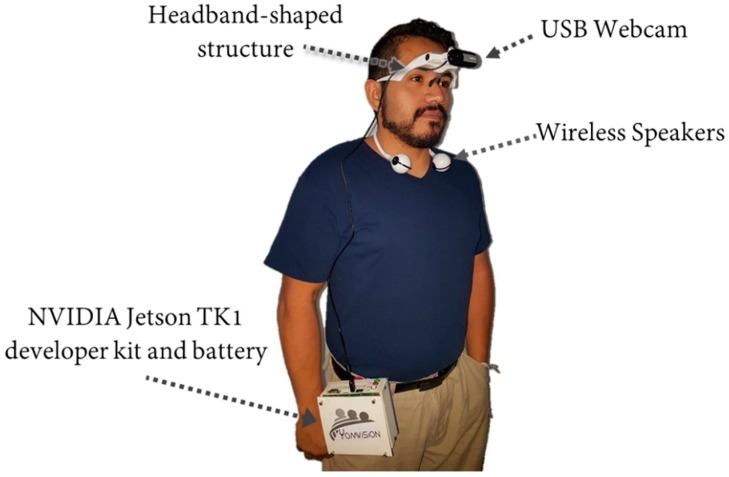
Integration of the face recognition system in real time which says aloud the name of the person identified.

When the model was correctly trained the next problem to solve was that during the process of face recognition the model indicates with a label that indicates to whom the face belongs, however, in case of having a visual impairment it is necessary that the name of the person who is identified be expressed aloud. For this purpose, loudspeakers are used, at this point, it is important to say that when using speakers and not hearing aids, it is possible for the disabled person not to use the sense of hearing exclusively for recognition. As shown in Figure 18, the speakers are conveniently placed in the neck with a flexible extensible, for this part of the process we made use of the synthetic voice of the loquendo software.

During the tests it was proved that the system was able to recognize in real time the people who were previously learned by the hybrid model Amαβ-CMKNN, the system also showed little sensitivity to facial expressions as it continued to recognize correctly. However, in future work, it will be necessary to create a learning mechanism for new faces which can be easily added by the user since it is normal that new people are known throughout our lives.

## 5. Discussion and Conclusions

The recognition of faces is a multifactorial task, thus a strategy to follow is to divide the problem by attacking the different factors that directly affect the performance of the algorithms when trying to recognize people under real conditions. The use of Amαβ-CMKNN to solve the problem of recognition when the face presents different facial expressions were considered. Two experiments were designed to evaluate the performance of a hybrid model in the task of recovery and classification. In both experiments, the CK+, CAS-PEAL-R1, and Face-MECS databases were used. For the detection and segmentation of the face in an image, the HLF technique was used, while the extraction of characteristics was performed with AAM. Later, the Amαβ-CMKNN was used for the recognition of faces.

In Experiment 1, 103 distances that generated characteristic binary patterns were extracted, with the distances being calculated from the 42 points that frame the elements that define the face. In Experiment 2, fewer features were used than in Experiment 1, and the patterns were composed of 13 ratios calculated from 16 distances. In both experiments, the memory was able to recall 100% of the patterns learned during the 10 rounds of the recovery phase. The results correspond to the fact that the self-associative memories were essentially designed to store information and efficiently retrieve it using the maximum (⋁) and minimum (⋀) operators.

During the test phase, the results show that the yields of all the models evaluated, including the Amαβ-CMKNN and the state of the art, show better results using the characteristics of Experiment 2, this is due to the fact that the extracted ratios were carefully selected as parametric features of the face have less sensitivity at the moment of deforming the face with some facial gesticulation or in rotation of the face with respect to the camera, the latter is closely related to the alignment process proposed in point detection with AAM.

The performances of the Amαβ-CMKNN with the CK + database was the best recorded with an average accuracy rate of 95.05%, this database presents faces with different facial expressions, however, it does not present different illuminations and face rotation. The Face-MECS database is a proprietary database created with videos captured with the system proposed in this paper, this was done in outdoor environments, thus that the automatic recognition of the face presents challenges of variation of illumination, facial expressions instantaneous, and face rotation among other factors, The reported results of the proposed Amαβ-CMKNN are an average accuracy rate of 86.48%, which compared to the state-of-the-art framed models demonstrates the competitiveness of the proposed hybrid model, surpassing the performance of the original model of the Amαβ and KNN, nevertheless, it is also possible to observe that the artificial neuronal network competes strongly with the proposed model.

Facial expressions are a natural process of non-verbal communication of the human being, however, the deformation of the face represents a challenge, since the models learn distances or facial relationships that are affected by the gestures. When analyzing the failures committed by the proposed hybrid model it is possible to say that the expressions of surprise, anger, and sadness are in which the model had greater complication to determine to whom a face belongs, this is because when the face expresses surprise it is it manifests the maximum deformation of the face, while in the anger and sadness the area of the eyes undergoes important deformations. On the other hand, when the face expressed happiness, the best results were obtained, this is in accordance with that reported by Yacoob and Davis [59] where it was concluded that the way of smiling has personalized features which allow it to be a strong characteristic of recognition to determine who the smile belongs to.

Therefore, and based on the results obtained when comparing the performance of Amαβ-CMKNN with other classifiers with equal conditions, one of the classifiers with which it was compared is the original model Amαβ and also KNN with Euclidean distance, showing better results than its predecessors, thus it is possible to say that the Amαβ-CMKNN is a model that presents competitive results, even when the faces are deformed by facial expressions. This in comparison with other classifiers that are usually also used in facial recognition.

Finally, the implementation of the hybrid model is shown in a system that integrates different devices to be used by people suffering from visual impairment or prosopagnosia, during the field tests it is possible to observe the advantages and weaknesses of the system, thus it is possible to observe that the devices that make up the system are functional, since they do not reduce the environmental auditory function, due to the use of wireless speakers and not hearing aids to listen to who owns the face, and the proposed speakers are lightweight and have a comfortable design for use around the neck. The Headband-shaped structure is also lightweight and does not interfere with the visual field, in addition, the autonomy time is between 2 and 2.5 h. Although in future works it is suggested to integrate mechanisms that allow the user to easily integrate new faces that the Amαβ-CMKNN model must learn. Additionally, it is necessary to integrate techniques to minimize the sensitivity of the system to changes in lighting.

## Figures and Tables

**Table 1:** Binary operators α and β.

A α A→B			B β A→A		
x	y	α(x,y)	x	y	β(x,y)
0	0	1	0	0	0
0	1	0	0	1	0
1	0	2	1	0	0
1	1	1	1	1	1
			2	0	1
			2	1	1

**Table 2:** Patterns contained in the training and test set using k-fold cross validation with *k* = 10.

Database	Training Set Patterns	Test Set Patterns	Rounds	Total Patterns Tested
CK+	1728	192	×10	1920
CAS-PEAL-R1	12,168	1352	×10	13,520
Face-MECS	21,870	2430	×10	24,300
